# Molecular Mechanism for Human Sperm Chemotaxis Mediated by Progesterone

**DOI:** 10.1371/journal.pone.0008211

**Published:** 2009-12-08

**Authors:** Maria E. Teves, Hector A. Guidobaldi, Diego R. Uñates, Raul Sanchez, Werner Miska, Stephen J. Publicover, Aduén A. Morales Garcia, Laura C. Giojalas

**Affiliations:** 1 Centro de Biología Celular y Molecular, Universidad Nacional de Córdoba, Córdoba, Argentina; 2 Centro de Biotecnología de la Reproducción, Departamento de Ciencias Preclínicas, Universidad de La Frontera, Temuco, Chile; 3 Centre for Dermatology and Andrology, Justus Liebig University, Giessen, Germany; 4 School of Biosciences, University of Birmingham, Birmingham, United Kingdom; New Mexico State University, United States of America

## Abstract

Sperm chemotaxis is a chemical guiding mechanism that may orient spermatozoa to the egg surface. A picomolar concentration gradient of Progesterone (P), the main steroidal component secreted by the cumulus cells that surround the egg, attracts human spermatozoa. In order to elucidate the molecular mechanism of sperm chemotaxis mediated by P, we combine the application of different strategies: pharmacological inhibition of signaling molecules, measurements of the concentrations of second messengers and activation of the chemotactic signaling. Our data implicate a number of classic signal transduction pathways in the response and provide a model for the sequence of events, where the tmAC-cAMP-PKA pathway is activated first, followed by protein tyrosine phosphorylation (equatorial band and flagellum) and calcium mobilization (through IP_3_R and SOC channels), whereas the sGC-cGMP-PKG cascade, is activated later. These events lead to sperm orientation towards the source of the chemoattractant. The finding proposes a molecular mechanism which contributes to the understanding of the signal transduction pathway that takes place in a physiological process as chemotaxis.

## Introduction

One of the primary questions in reproductive biology concerns the mechanism(s) by which gamete encounter is achieved. Chemotaxis, the sperm travelling to the egg guided by a concentration gradient of a chemical attractant, was reported for several mammalian species towards fluids or cell conditioned medium from the egg microenvironment [1]. The main steroid from all these attractant sources is Progesterone (P), which is secreted by the cells that surround the egg and forms a stable concentration gradient along the cumulus oophorus [2], [3]. This hormone induces sperm chemotaxis mainly at very low concentrations (pM), only in a ∼10% subpopulation of cells [4], [5]. Moreover, it seems to be the only physiological chemoattractant secreted by the cumulus cells [3]. Though sperm chemotaxis signaling has been partially described for external fertilizing species like sea urchin, starfish and ascidian [1], in mammalian sperm it is poorly understood [1]. Bourgeonal, a chemoattractant for which no endogenous equivalent has yet been identified, has been proposed to stimulate olfactory receptors (hOR17-4), leading to activation, via G_olf_ of tmAC and Ca^2+^ influx [6].

For the study of chemotactic signal transduction the spermatozoon is a limited cellular model since it is believed to be a highly differentiated transcriptionally inactive cell, where most of the molecular approaches cannot be applied (e.g. transfection, protein over expression, etc). Thus, the use of specific inhibitors of signaling pathways has been the most widely used approach, but providing limited information. Therefore, to study the molecular mechanism of human sperm chemotaxis towards P, we combined different strategies which involve: the suppression of chemotaxis with specific inhibitors of signaling molecules, the determination of intracellular level of second messengers, and the activation of the chemotactic signaling pathways by increasing the cyclic nucleotide intracellular level.

## Results

### The AC-cAMP-PKA Pathway

**Figure 1:**
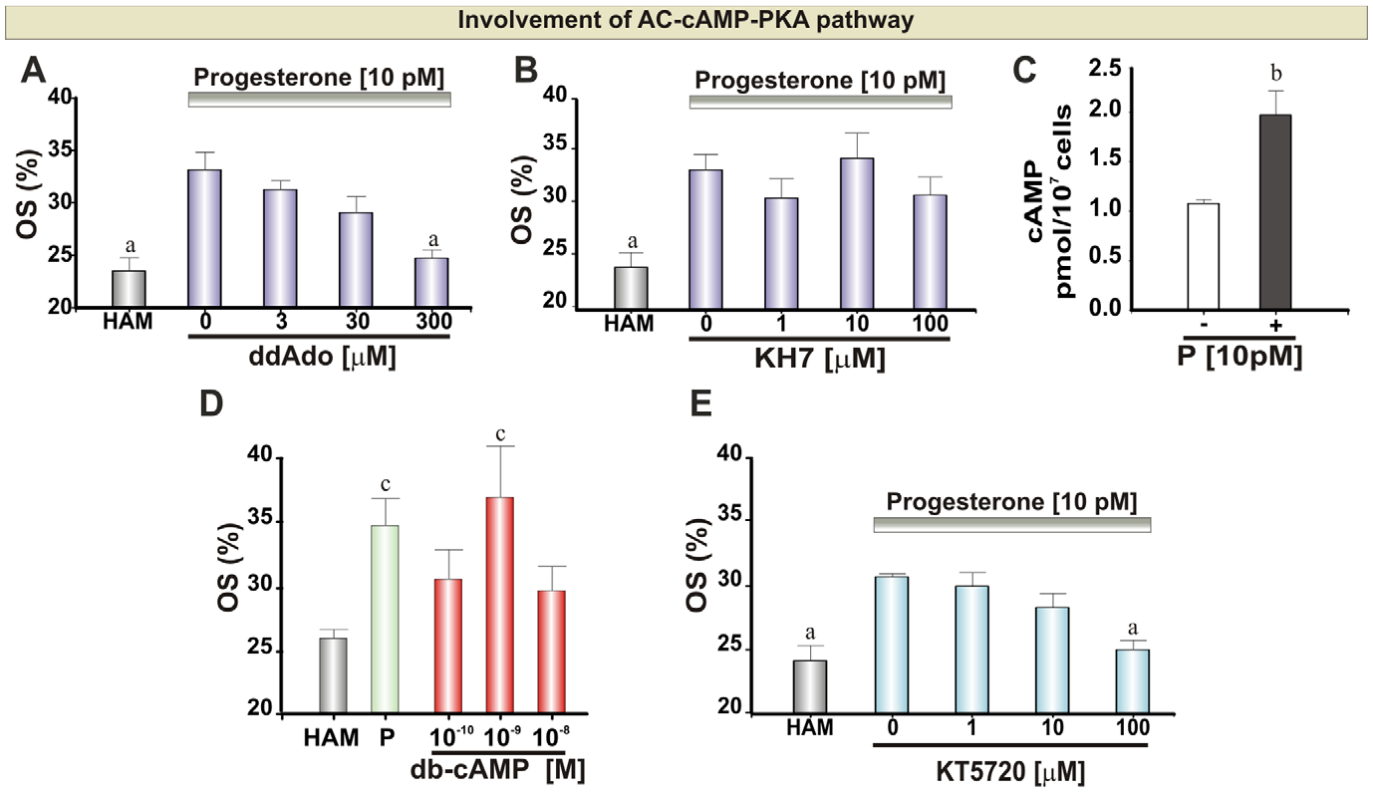
AC-cAMP-PKA pathway during sperm chemotaxis mediated by P. **A**, **B** and **E**: Percentage of oriented spermatozoa (OS) towards 10 pM P gradient after treating the cells with ddAdo (15 min), KH7 (15 min) and KT5720 (5 min), respectively. **C**: cAMP intracellular concentration in the absence or presence of 10 pM P. **D**: Percentage of oriented spermatozoa towards db-cAMP. HAM was assayed as a negative control. Each bar represents the mean ± SEM. ^a^ Significant differences vs. without inhibitor (p<0.05). ^b^ Significant differences vs. without P (p<0.05). ^c^ Significant differences vs. HAM (p<0.05).

Adenylyl Cyclase (AC) is expressed in mammalian spermatozoa in both soluble (sAC) [7], [8] and transmembrane (tmAC) [6], [9]–[11] forms. First, we exposed spermatozoa to a gradient of 10 pM P after a pre-treatment with several concentrations of ddAdo or KH7, inhibitors of tmAC and sAC, respectively. A complete inhibition of chemotaxis (but not motility) was observed at 300 µM ddAdo (Figs. 1A and S1A), consistent with the potency of this compound in other systems [12]. In contrast, KH7 at concentrations up to 100 µM, did not affect P-induced chemotaxis or motility (Figs. 1B and S1B). To confirm the activity of KH7 we assessed its efficacy in inhibiting acrosome reaction (AR), which is dependent on sAC activation in both sea urchin [13] and human [14] spermatozoa. AR induced by A23187 was indeed inhibited in a dose dependent way by KH7 (Fig. S1D). Since AC appear to be involved in chemotaxis, we further investigated whether there was an increase in the second messenger cAMP upon stimulation with chemotactic doses of P. A significant increase in cAMP concentration was observed after incubating the cells with 10 pM P (Fig. 1C). In addition, to dissect the participation of cAMP we evaluated the activation of the chemotactic signaling by means of a gradient of db-cAMP, a membrane permeant cAMP analog (see experiment rationale below). The activation of the chemotactic cascade was observed under a gradient generated by 10^−9^ M db-cAMP (Fig. 1D and S5A). As a consequence of an increase in intracellular cAMP concentration, Protein Kinase A (PKA) may be activated. Therefore, spermatozoa were exposed to a 10 pM P gradient after pre-treatment with a PKA inhibitor (KT5720). Figure 1E shows that P mediated chemotaxis (but not motility; Fig. S1C) was completely abolished at high inhibitor doses (100 µM).

### The GC-cGMP-PKG Pathway

**Figure 2:**
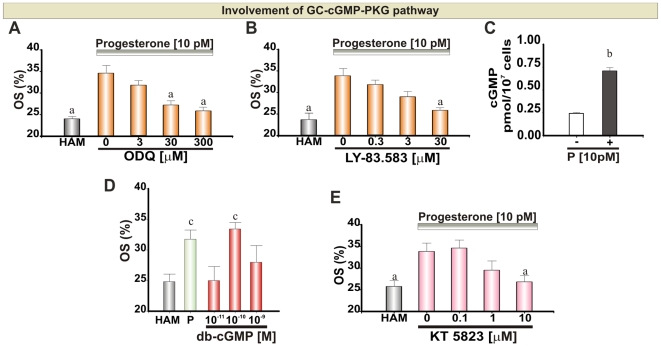
GC-cGMP-PKG pathway during sperm chemotaxis mediated by P. **A**, **B** and **E**: Percentage of oriented spermatozoa (OS) towards 10 pM P gradient after treating the cells with ODQ (15 min), LY-83.583 (15 min) and KT5823 (5 min), respectively. **C**: cGMP intracellular concentration in the absence or presence of 10 pM P. **D**: Percentage of oriented spermatozoa towards db-cGMP. HAM was assayed as a negative control. Each bar represents the mean ± SEM. ^a^ Significant differences vs. without inhibitor (p<0.05). ^b^ Significant differences vs. without P (p<0.05). ^c^ Significant differences vs. HAM (p<0.05).

Guanylyl Cyclase (GC) is expressed in sperm as a membrane (mGC) and a soluble guanylyl cyclase (sGC) [15]. Since mGC is a well characterized surface receptor for ligands different from P [15] we analyzed only the involvement of sGC in the chemotactic signaling mediated by P. Cells were pre-treated with two sGC inhibitors (ODQ and LY-83.583). A dose-dependent inhibition of P–mediated chemotaxis was observed with a maximum at 30–300 µM ODQ and 30 µM LY- 83.583 (Figs. 2A and B), with no significant effect on sperm motility (Figs. S2A and B). In addition, to verify whether chemotactic doses of P activate cGMP signaling, we treated spermatozoa with 10 pM P, where a significant increase in the level of intracellular cGMP was observed (Fig. 2C). Moreover, we activated the chemotactic signaling by means of a gradient of db-cGMP, a membrane permeant cGMP analog (see experimental rationale below). The activation of the chemotactic cascade was observed under a gradient generated by 10^−10^ M db-cGMP (Fig. 2D and S5B). Protein Kinase G (PKG) activation depends on the increase of cGMP, therefore, spermatozoa were pre-treated with a PKG inhibitor. Sperm chemotaxis was inhibited by 10 µM KT5823 (Fig. 2E), whereas motility was not affected (Fig. S2C).

### Calcium as a Second Messenger

**Figure 3:**
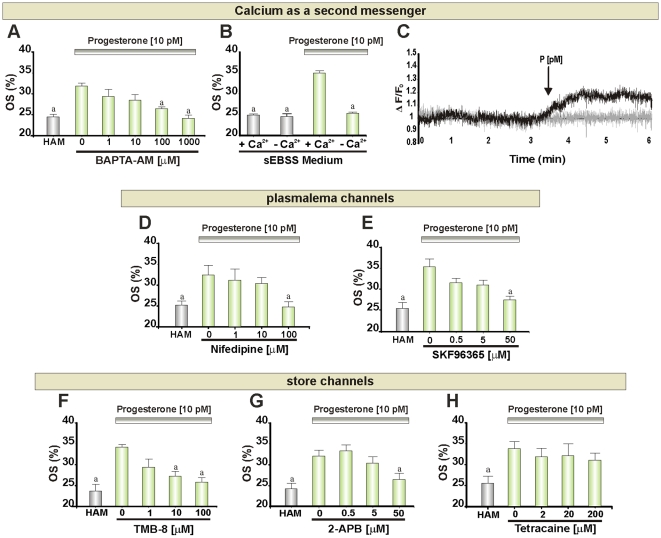
Calcium as a second messenger for sperm chemotaxis. **A**, **B**, **D**, **E**, **F**, **G** and **H**: Percentage of oriented spermatozoa (OS) towards 10 pM P gradient after treating the cells with BAPTA-AM (1 min), sEBSS culture medium with and without extracellular calcium, Nifedipine (1 min), SKF96365 (1 min), TMB-8 (1 min), 2-APB (15 min) and Tetracaine (15 min), respectively. **C**: Ca^2+^ signal (change in Oregon green 1 BAPTA florescence) in immobilized human spermatozoa, black line shows the mean of cells that responded to a step application of pM P and gray line shows the mean of those cells in which no response was detectable. Black arrow marks time of arrival of P in the imaging chamber. HAM was assayed as a negative control. Each bar shows mean ± SEM. ^a^ Significant differences vs. without inhibitor (p<0.05).

Calcium signaling plays a key role in sperm physiology [1], [16], [17], and it is well established that P at nM-µM concentrations can elevate [Ca^2+^]_i_ in mammalian sperm. When cells were pre-treated with BAPTA-AM to prevent generation of Ca^2+^ signals in the cytoplasm, chemotaxis was completely inhibited by 100 µM of the calcium chelator concentration (Fig. 3A). No significant effect on sperm motility was observed during the assay (Fig. S3A). In addition, to verify whether chemotactic doses of P generate calcium signaling, immobilized spermatozoa were labeled with Oregon Green BAPTA-1 AM and exposed to a 10 pM P step, where an increase in [Ca^2+^]_i_ in a sperm subpopulation (15±3%) was observed (Fig. 3C).

#### Ca^2+^ influx and plasma membrane Ca^2+^ channels

Buffering of [Ca^2+^]_o_ to submicromolar concentrations with BAPTA or EGTA had marked effects on cell motility, making assessment of P-induced chemotaxis impossible. Therefore, to address the involvement of calcium influx we used sEBSS medium without added Ca^2+^ salt, which reduces [Ca^2+^]_o_ to <10 µM [18]. Under these conditions sperm chemotaxis towards P was suppressed (Fig. 3B) without significant changes in motility (Fig. S3B). Then, we attempted to identify the nature of the Ca^2+^ channels that are activated during the chemotactic response. Pre-treatment with nifedipine, which inhibits both L-type (Ca_v_1) and T-type (Ca_v_3) channels that are believed to be expressed and physiologically important in mammalian spermatozoa [19], blocked P-induced chemotaxis in a dose dependent manner without affecting sperm motility (Fig. 3D; Fig. S3C), with complete inhibition at high 100 µM. In order to investigate whether store-operated calcium (SOC) channels might also be involved, we used SKF96365 to blockade of Ca^2+^ influx through SOC channels. 50 µM SKF96365 abolished the chemotactic response (Fig. 3E), without affecting sperm motility (Fig. S3D).

#### Ca^2+^ store mobilization and Ca^2+^ store channels

Cells were pre-treated with TMB-8, which inhibits stored Ca^2+^ mobilization in somatic cells [20]–[22]. This compound caused a clear, dose-dependent inhibition of chemotaxis that was statistically significant at 10 µM (Fig. 3F) and had no effect on cell motility (Fig. S3E). Furthermore, we used tetracaine to inhibit ryanodine receptors (RyRs), present in the neck Ca^2+^ store membrane and 2-APB to block inositol 1,4,5-trisphosphate receptors (IP_3_Rs), which are believed to be present in both the membrane of the acrosomal and neck Ca^2+^ store [23]. Tetracaine, up to 200 µM, had no effect in P-mediated chemotaxis (Fig. 3H) and at 2 mM the cells were non-motile (Fig. S3G) and dead. In contrast, the blockade of IP_3_R channel with 50 µM 2-APB abolished sperm chemotaxis (Fig. 3G) without changing sperm motility (Fig. S3F).

### Protein Tyrosine Phosphorylation

**Figure 4:**
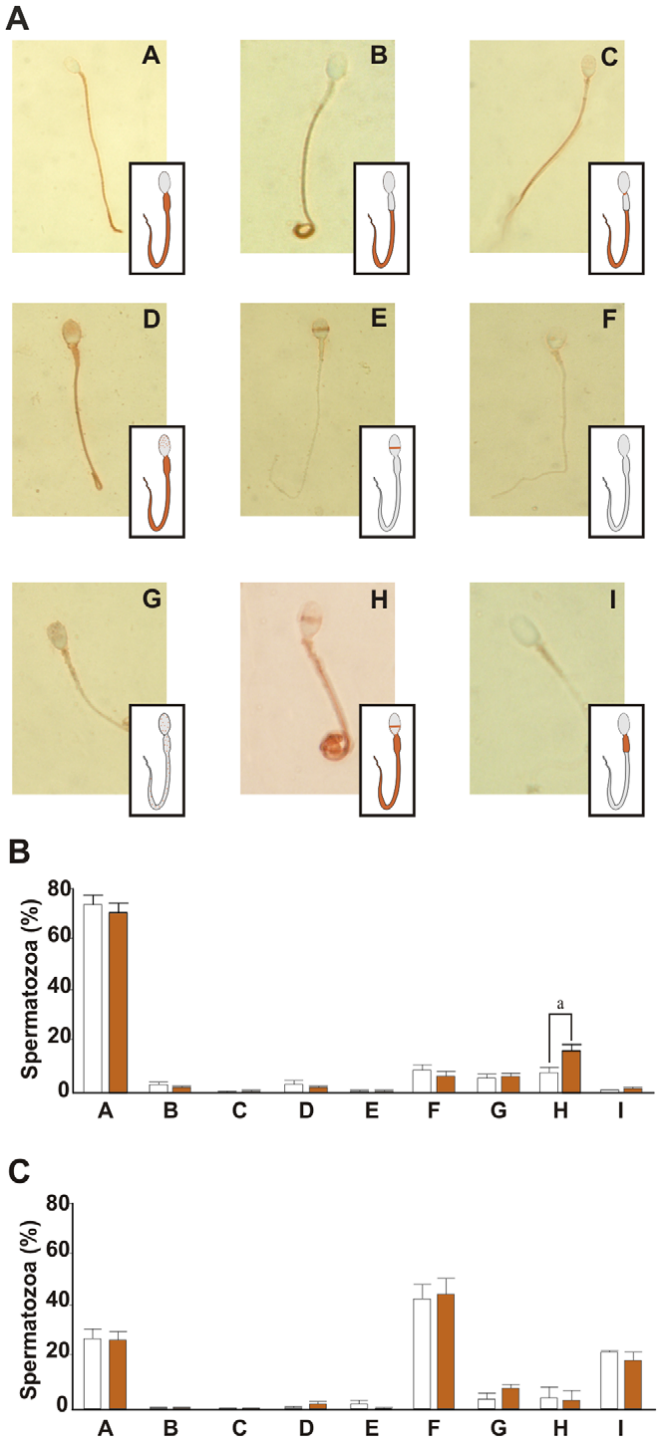
Sperm protein tyrosine phosphorylation. **A**: Immunocytochemistry for protein Tyr-phosphorylation in the absence or presence of a 10 pM P gradient. ^A^ flagellum; ^B^ principal and end piece; ^C^ neck, principal and end piece; ^D^ punctuate staining of the acrosome region and flagellum; ^E^ equatorial band; ^F^ no label; ^G^ punctuate staining of the whole spermatozoon; ^H^ equatorial band and flagellum; ^I^ midpiece. Percentage of each identified pattern of Tyr-phosphorylation after treating the cells with (dark) or without (white) 10 pM P gradient in: (**B**) spermatozoa incubated under capacitating or (**C**), non-capacitating conditions. Data are expressed as the mean ± SEM. ^a^ Significant differences vs. without P (p<0.05).

Tyrosine phosphorylation of flagellar proteins, occurring during sperm capacitation, is believed to regulate sperm motility [24]. In order to test whether protein Tyr-phosphorylation was associated with sperm chemotaxis, immobilized live spermatozoa were exposed to a gradient of 10 pM P in a chemotaxis chamber and then assessed for protein Tyr-phosphorylation by immunocytochemistry. Nine patterns of phosphotyrosine staining (A–I) were identified in spermatozoa (Figure 4A), where most of the cells exhibited pattern “A”, which is characterized by labeling throughout the flagellum (Figure 4B). After exposure to a P gradient the distribution of the staining patterns was largely similar except that there was a significant increase (∼10%; similar to the percentage of chemotactic spermatozoa) in the proportion of cells showing pattern “H” (Figure 4B), characterized by labeling in the equatorial band and flagellum. To further investigate whether the observed pattern “H” was a consequence of AR, the cells were double-labeled with the anti-phosphotyrosine antibody in combination with an anti-proacrosin (anti-Rec30) antibody which distinguishes acrosome reacted from acrosome intact spermatozoa [25] (Figure S4). The results showed that pattern “H” is observed only in acrosome intact spermatozoa (Figure S4B) in agreement with the report of Tomes et al (2004) [26]. In order to evaluate whether the P-induced increase in pattern “H” was dependent upon sperm capacitation, these experiments were repeated with spermatozoa treated in the absence of albumin, condition under which capacitation is greatly reduced [27], [28]. Although, all nine patterns of protein Tyr-phosphorylation were observed in these cells, the proportion of cells expressing each pattern was markedly different, patterns “F” and “I” being greatly increased in abundance (Figure 4C). However, no differences were observed in cells exposed to a chemotactic gradient of P (Figure 4C). In addition, an increase in protein Tyr-phosphorylation mediated by a gradient of 10 pM P was also observed by SDS PAGE-Western Blot (data not shown).

### Sequence of Chemotactic Signaling Events

**Figure 5:**
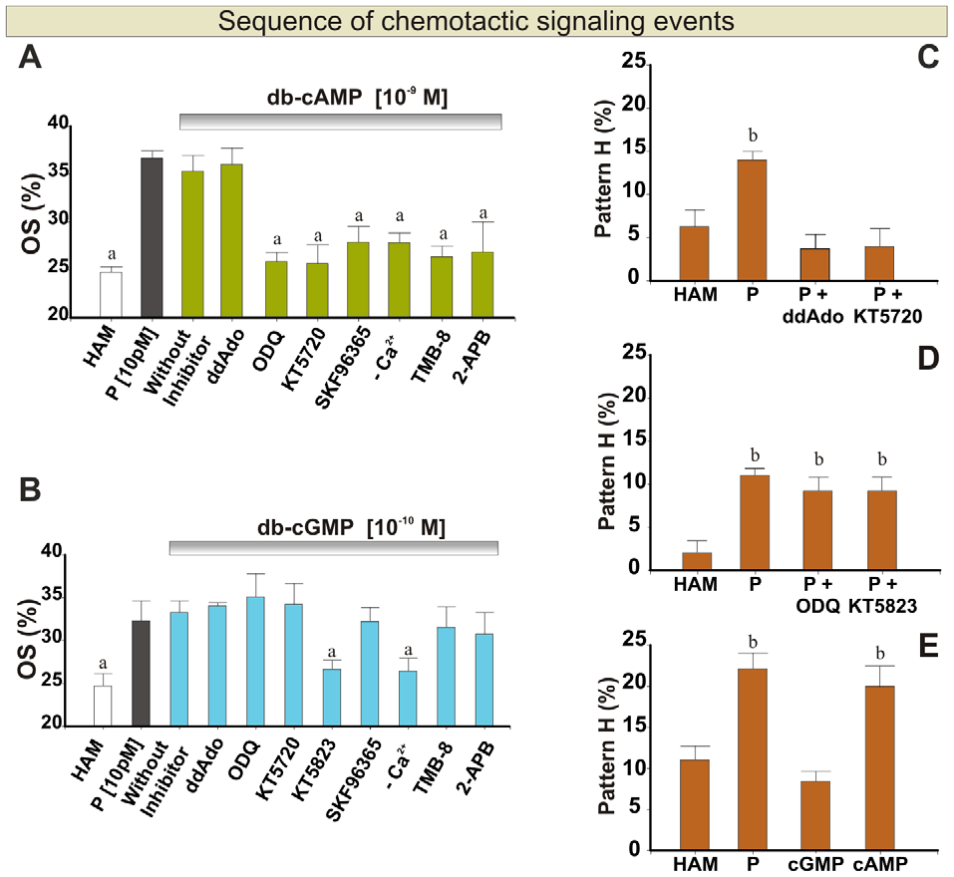
Sequence of chemotactic signaling events. **A** and **B**: Percentage of oriented spermatozoa (OS) towards: (**A**) 10^−9^ M db-cAMP or (**B**) 10^−10^ M db-cGMP gradient in the presence of effective doses of the following inhibitors ddAdo (300 µM), ODQ (30 µM), KT5720 (100 µM), SKF96365 (50 µM), sEBSS medium without calcium, TMB-8 (10 µM); 2-APB (50 µM) and KT5823 (10 µM). **C** and **D**: Percentage of spermatozoa showing protein Tyr-phosphorylation pattern “H” after exposure to 10 pM P gradient and pre-treated with (**C**) ddAdo (300 µM) or KT5720 (100 µM) inhibitors and (**D**) ODQ (30 µM) or KT5823 (10 µM) inhibitors. **E**: Percentage of cells showing pattern “H” after exposure to 10 pM P, 10^−9^ M db-cAMP or 10^−10^ M db-cGMP gradient. HAM was assayed as a negative control. Data are expressed as the mean ± SEM. ^a^ Significant differences vs. without inhibitor (p<0.05). ^b^ Significant differences vs. HAM (p<0.05).

In the results described above, we observed that tmAC-cAMP-PKA, sGC-cGMP-PKG, Ca^2+^ mobilization and protein Tyr-phosphorylation may be involved in the P-mediated chemotactic signaling. However, those results did not give information on the sequence of molecular events. To study the molecular mechanism we need to activate the sperm chemotactic signaling bypassing the binding of P to its receptor (P_R_). Considering that the chemotaxis cascade involves the increase in cyclic nucleotide concentration (Fig. 1C and 2C), we used extracellular gradients of cell-permeable cyclic nucleotide analogs to mimic the action of P on sperm chemotaxis, as a consequence of an increase in the intracellular concentration of second messengers. This rationale is based on observations made in other chemotactic models, where extracellular gradients of db-cAMP and db-cGMP where used to stimulate the chemotactic cascade that promote the growth of the axon cone [29]. Such cyclic nucleotide extracellular gradients induce intracellular gradients of second messengers in microdomains (≈1–5 µm) of the axon cone, of similar size to the sperm head. Then, we first defined the concentration of db-cAMP and db-cAMP, necessary to stimulate a significant increase in the percentage of oriented spermatozoa by activating the cascade downstream the P binding (Fig. 1D and 2D). We studied the sequence of molecular events stimulating spermatozoa with these active concentrations of the analogs, and in combination with inhibitors of the molecules that seems to participate in the P-mediated chemotactic response. Thus, in the case that chemotaxis signaling is suppressed in the presence of an inhibitor, it means that the blocked molecule participates downstream the increase in the cyclic nucleotide. On the contrary, if chemotaxis signaling activation is not abolished, it means that the inhibited molecule acts upstream the increase in the cyclic nucleotide. Figure 5A shows that the activation of chemotactic response by a gradient of db-cAMP was blocked when cells were pre-treated with the effective doses of inhibitors of sGC, PKA, stored Ca^2+^ release, SOC and also in saline with reduced [Ca^2+^]_0_, whereas no block was observed under tmAC inhibition, as expected. In contrast, the chemotactic response activated by a gradient of db-cGMP was blocked only after inhibiting PKG or reduction of [Ca^2+^]_0_ in the medium (Fig. 5B), whereas no block was observed under the inhibition of sGC, tmAC-cAMP-PKA pathway, and calcium mobilization. These results suggest a sequence for the signaling cascade where the first steps would be the activation of tmAC-cAMP-PKA pathway, followed by release of stored Ca^2+^ and Ca^2+^ influx through SOC, and sGC activation. Moreover, after the increase in the cGMP level there may be a second Ca^2+^ influx and the PKG become activated. Supplementary table S1 summarizes which molecular events could take place upstream or downstream the respective cyclic nucleotide signaling activation. In addition, these results also suggest that the inhibitors are specific, since inhibition was observed only in some steps of the cascade, but not in all of them.

In order to know whether proteins are phosphorylated in tyrosine as a consequence of the activation of protein tyrosine kinases (PTK) downstream AC-cAMP-PKA or GC-cGMP-PKG pathway, we first evaluated the occurrence of pattern “H” when the cells were stimulated with 10 pM P in the presence of specific inhibitors. There was a significant decrease in the percentage of cells showing pattern “H” only when the tmAC or PKA were inhibited (Figs. 5C and D). To further verify the latter result, spermatozoa were exposed to an extracellular gradient of db-cAMP or db-cGMP, where a significant increase in the percentage of cells with the specific pattern “H” was observed only upon stimulation with the cAMP analog, and at the same level as observed with 10 pM P (Fig. 5E).

As a whole, these results suggest a sequence for the chemotactic signaling activation starting with the tmAC-cAMP-PKA pathway, followed by protein Tyr-phosphorylation, calcium mobilization, and the activation of sGC-cGMP-PKG cascade, with a later second calcium influx.

## Discussion

During the last decade, it became clear that sperm chemotaxis is a necessary guidance mechanism for transporting spermatozoa to the egg in mammals. We recently showed that tiny amounts of P secreted by the cumulus cells chemoattract human and rabbit spermatozoa at pM concentrations [2], becoming the cumulus oophorus and its surrounding the probable site for sperm chemotaxis *in vivo* [2], [3]. In the present study we used a combination of different strategies: 1) by inhibiting specific signaling molecules, we first showed the possible participation of two cascades: tmAC-cAMP-PKA and sGC-cGMP-PKG in addition of calcium mobilization; 2) by determining the intracellular level of second messengers, we verified whether the respective cascades and Ca^2+^ mobilization were involved in sperm chemotaxis; 3) by activating the chemotaxis signaling with cyclic nucleotide analogs that pass through the sperm membrane, we deciphered the probable sequence of signaling events that may take place during sperm chemotaxis.

As a whole, these tools allowed us to propose, for the first time, a tentative model for the molecular mechanism of sperm chemotaxis mediated by a physiological attractant, progesterone.

### tmAC-cAMP-PKA Signaling

We first showed that activation of tmAC may be required during sperm chemotaxis mediated by P since the chemotactic effect was lost in the presence of the inhibitor ddAdo, whereas inhibition of sAC had no detectable effect on chemotaxis. Taking into account that the use of inhibitors must be interpreted cautiously, to further dissect the involvement of cAMP we measured the intracellular levels of this nucleotide, where an increase in the cAMP concentration upon chemotactic doses of P was observed. In addition, the chemotactic pathway was stimulated by a membrane permeant cAMP analog (db-cAMP), which may activate the chemotactic pathway bypassing the P binding to its receptor. Our conclusion on the effect of ddAdo on P-induced chemotaxis is due to a specific inhibition of the generation of cAMP is supported by the failure of the drug to inhibit the effect of db-cAMP (Fig. 5A). It is interesting to note that chemotactic signaling activation effects of db-cAMP were seen with a <1 nM gradient, but not at higher doses (µM-mM) where this compound is commonly used by others to induce sperm processes like capacitation [30], acrosome reaction [31] and protein phosphorylation [32]. It is not surprising that 1 nM concentrations of db-cAMP stimulate the chemotactic signaling since the latter process can be induced by pM P, consistent with the dose response increase of cAMP observed by Parinaud et al. (1996) [33] upon increasing concentrations of P.

Additionally, our results showed that chemotaxis was suppressed by inhibiting PKA with KT5720. It is noteworthy that the active concentration of this inhibitor was relatively high (100 µM) in comparison to the effective doses observed in other cellular models (1–10 µM) [34], [35]. Interestingly, the exposure of spermatozoa to a P gradient induces an increase in the proportion of cells showing protein Tyr-phosphorylation in the equatorial band and flagellum, in a similar location reported for PKA in mouse spermatozoa [36].

As a whole, the activation of the tmAC-cAMP pathway (and possibly PKA) is apparently an important step in P-induced chemotaxis, similarly to the observations made on spermatozoa stimulated with bourgeonal [6].

### sGC-cGMP-PKG Signaling

Our experimental strategy suggested the involvement of sGC in P-mediated chemotactic signaling indicated by two different inhibitors of the enzyme (ODQ and LY-83.583) acting at similar doses as reported by others [37], [38]. Additionally, the specificity of ODQ was verified in the experiments carried out to study the sequence of molecular events (Fig. 5B). However, to further investigate whether cGMP is a necessary component of human sperm chemotaxis we measured the intracellular levels of this nucleotide, where there was a significant increase in the cGMP level when cells were incubated in the presence of 10 pM P. In addition, the chemotactic pathway was activated by db-cGMP. The chemotaxis activation observed under 0.1 nM db-cGMP is consistent with observations made in sea urchin spermatozoa where one attractant molecule may stimulate the synthesis of ∼5 cGMP molecules [16].

The cGMP-activated kinase PKG appears to be implicated in the P-mediated chemotaxis signaling since the inhibitor (KT5823) suppressed the sperm chemotactic response at an expected dose [39]. The cGMP-PKG pathway has also been proposed to underlie human sperm chemotaxis in response to NO donors [38]. Though, NO is secreted by the cumulus cells [40], it is unlikely to form a gradient along the cumulus (necessary for chemotaxis *in vivo*), since it is a high diffusible molecule with a short lifetime. Although Miraglia et al. (2007) [38] used similar sGC inhibitor doses, the methods used for chemotaxis evaluation do not distinguish the latter sperm process from others causing cell accumulation.

The activation of sGC-cGMP-PKG pathway is apparently necessary for human sperm chemotaxis, in agreement with the chemotactic signaling reported for sea urchin spermatozoa [16].

### Calcium Signaling

Calcium plays a key role in many functions of both invertebrate and vertebrate sperm and is central to recent models of chemotaxis in sea urchin [16], ascidia [41] and human sperm [6]. We initially investigated the participation of Ca^2+^ in P-mediated chemotaxis by pretreating cells with BAPTA-AM to prevent generation of the cation signals induced by P, which effectively prevented P-mediated chemotaxis. In addition, we observed that human spermatozoa exposed to a chemotactic dose of P, increased the [Ca^2+^]_i_. Moreover, when Ca^2+^ was simply omitted from the bathing medium, the chemotactic response to P was significantly inhibited. To further investigate this Ca^2+^ dependence, we assessed the effect of pre-treatment with a number of plasma membrane Ca^2+^ channel blockers. To investigate the possible role of Ca_v_ channels, which are known to be expressed in mammalian sperm [19], we used the blocker nifedipine. Sperm chemotaxis was suppressed when spermatozoa were pre-treated with the drug at 100 µM. It is noteworthy that the completely effective dose is 10x higher than that reported to decrease the sustained response of the transient calcium influx and AR in human spermatozoa stimulated by P [42], and 100 times greater than that required to inhibit T-type currents of mouse spermatogenic cells and sperm [43], [44]. It thus, appears unlikely that the effects of nifedipine reported here are due to effects Ca_v_ channels and we conclude that their participation in P-induced chemotactic signaling is unlikely. CNG type Ca^2+^-permeable channels have also been described in mammalian spermatozoa [45], [46] and might be activated downstream of AC or GC activation. However, these channels can be effectively blocked by tetracaine [47], which did not interfere with P-mediated chemotactic response (see below), thus it seems that CNG may not be responsible for Ca^2+^ influx during chemotaxis mediated by P. In contrast, the SOC channel blocker SKF96365 inhibited human sperm chemotaxis at a doses consistent with its effects on SOCs, similarly to the reported block of chemotaxis in ascidian sperm [48]. Furthermore, the antagonist TMB-8 (known to inhibit both IP_3_R [20], [22] and RyR [21] calcium channels) significantly inhibited chemotaxis at 10 µM, a dose consistent with effects on store mobilization and modest compared to that reported to arrest intracellular calcium oscillations in human sperm stimulated by micromolar P [18]. Thus, it appears likely that store mobilization and activation of SOCs occurs during P-induced chemotaxis of human sperm and underlies at least part of the requirement for Ca^2+^ influx. Additionally, a failure of tetracaine to inhibit chemotaxis suggests that RyRs are not important in this process. In contrast, the ability of 50 µM 2-APB to inhibit chemotaxis is consistent with a role of IP_3_Rs for AR stimulated by micromolar P [49]. Since 2-APB also acts as an effective blocker of SOCs [50], the role for IP_3_Rs in chemotaxis should be interpreted with caution. However, results suggest that store mobilization and activation of SOCs may be important components of P-mediated chemotactic signaling. Progesterone at pM concentrations has been shown to induce Ca^2+^ release in the neck region of human sperm [51], suggesting that the redundant nuclear envelope (RNE) might be involved in P-mediated chemotaxis. In order to address which Ca^2+^ store is involved in chemotaxis (the acrosome or the RNE?) additional experiments should be done.

Our results suggest the need for calcium mobilization in the sperm chemotactic response, in agreement with the findings of other groups [6], [16].

### Protein Phosphorylation

Sperm immunolabeling for phosphotyrosine showed nine different staining patterns (A–I), similarly to previous reports [52]–[54]. After exposition to a gradient of 10 pM P a significant increase in the proportion of spermatozoa showing protein Tyr-phosphorylation in the equatorial band and flagellum (pattern “H”) was only observed in cells incubated under capacitating conditions. This result was not surprising since only capacitated spermatozoa are able to respond chemotactically to 10 pM P. In addition, the cells showing pattern “H” were acrosome intact. Thus, the increase in the proportion of cells with a protein Tyr-phosphorylation in the equatorial band and flagellum may be associated with the P-mediated chemotactic response and neither with capacitation nor acrosome reaction signaling.

Further experimentation with specific inhibitors in combination with cyclic nucleotide analogs showed that the increase in the phosphorylation pattern “H” was mediated by the activation of cAMP but not cGMP pathway during chemotactic signaling.

### Model for the Molecular Mechanism of Sperm Chemotaxis

Most of the knowledge on sperm chemotactic signaling pathway comes from studies in marine invertebrates. In human spermatozoa there is some evidence concerning the involvement of AC/cAMP/calcium [6] and GC/cGMP [38] in response to an attractant different from P, whereas the precise molecular mechanism is not defined yet. We propose a tentative model to explain the molecular events that may take place during human sperm chemotaxis upon stimulation with a pM gradient of P. The model is based on an experimental design which involves the activation of the signaling cascade with extracellular gradients of cyclic nucleotide analogs in combination with inhibitors. Here, sperm chemotaxis is achieved bypassing the binding of P to its receptor, whereas the analogs do not act as putative chemoattractant, but instead cross the cell membrane increasing the intracellular concentration of cyclic nucleotides, which in turn activate the chemotactic signaling. A similar approach was used to study signaling events of axon cone growth mediated by chemotaxis [29], [55]–[57].

**Figure 6:**
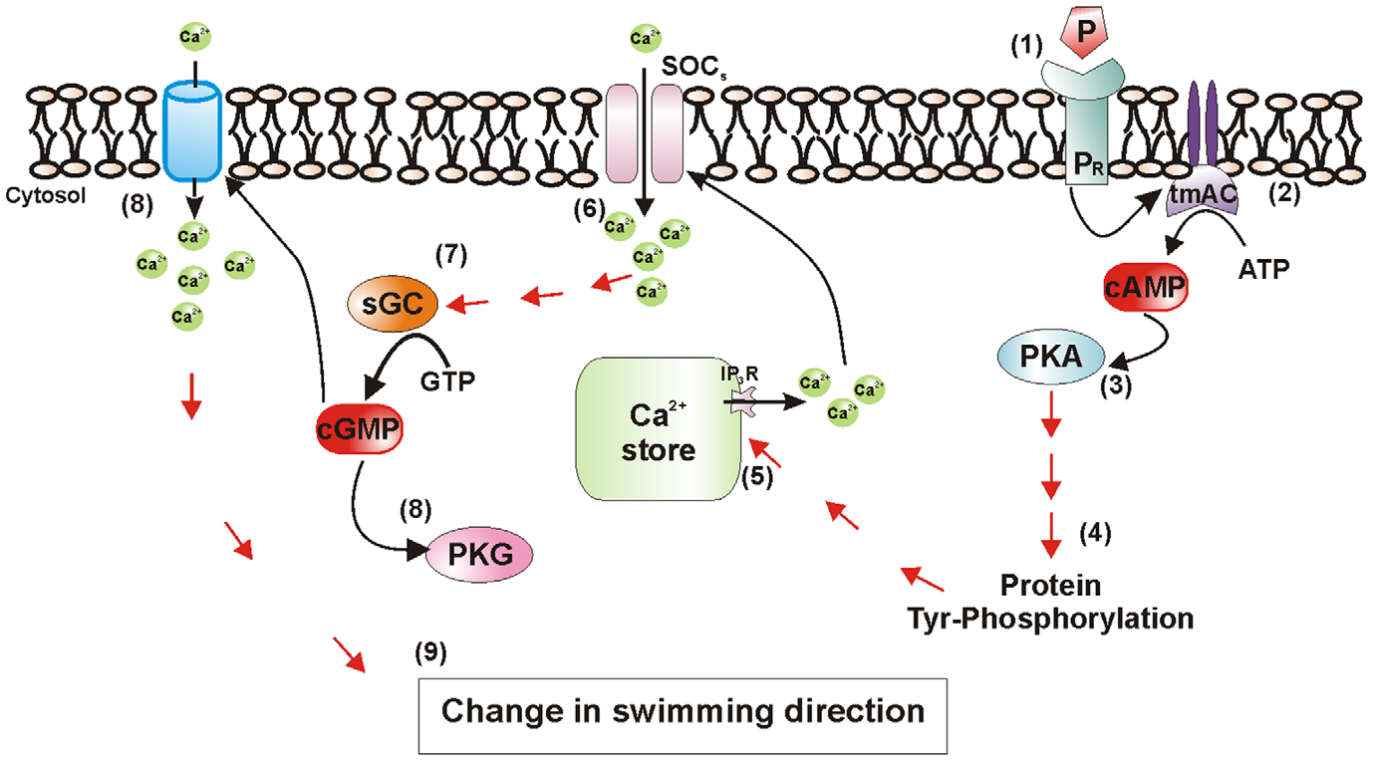
Molecular mechanisms involved in the sperm chemotaxis mediated by progesterone. 1, P-P_R_ interaction; 2, tmAC activation and cAMP synthesis; 3, PKA activation; 4, protein phosphorylation; 5, stored Ca^2+^ released through IP_3_R channel; 6, first Ca^2+^ influx through SOC channel; 7, activation of sGC with an intracellular cGMP increase; 8, PKG activation and a second Ca^2+^ influx probably through other calcium channels; 9, change in sperm swimming direction. Data from this study are shown with black arrows while unknown connecting steps are marked with red arrows. See text for further explanation of the model.

Under this experimental design we identified which molecules were activated upstream or downstream the respective cyclic nucleotide signaling activity (Supplementary table S1). Thus, after the binding of P to its cell surface receptor, the tmAC-cAMP-PKA pathway is activated first, followed by protein tyrosine phosphorylation (equatorial band and flagellum) and calcium mobilization (through IP_3_R and SOC channels), whereas the sGC-cGMP-PKG cascade, is activated at a later time, and possibly further Ca^2+^ influx through another plasma membrane calcium channels occur. These sequential steps would favor sperm orientation towards the P source (Fig. 6).

Our model is tentative, and many questions are still to be addressed. For instance, which are the other steps in the chemotaxis signal transduction?, what is the identity of the flagellar proteins that produce the orientation towards the attractant source?, which other molecules and ion channels are involved?. The answers to these questions will enlarge the understanding of mammalian sperm chemotaxis mediated by P, which in turn may be used not only to improve the assisted reproduction procedures for humans and animals, but also for the study of chemotactic molecular signaling in other cellular models.

## Materials and Methods

### Ethics Statement

The protocol for handling human sperm samples has been approved by the Bioethics Committee at the Centre for Cellular and Molecular Biology from National University of Cordoba, Argentina. All participants gave written informed consent.

### Reagents

HAM F-10 medium with L-glutamine and 25 mM Hepes (Invitrogen, USA); Human Serum Albumin (UNC, Argentina); Nifedipine, 2-APB (Calbiochem, Germany); KH7 (ChemDiv, USA); mouse monoclonal anti-phosphotyrosine, clone 4G10 (UPSTATE, USA); rabbit polyclonal anti-Rec30; fluorescent mounting medium (DAKO, Denmark); Poli-L-lysine (BIOCHROM AG, UK); Poli-D-lysine (BD Bioscience, UK); Streptavidin-Peroxidase (LAB-VISION, UK); cGMP and cAMP Enzymeimmunoassay Biotrak (EIA) System (Amersham Biosciences, UK); db-cAMP, db-cGMP, ddAdo, ODQ, KT5823, KT5720, SKF96365, LY-83.583, TMB-8, Tetracaine, BAPTA, BAPTA-AM, calcium ionophore A23187, Biotin-conjugated anti-mouse, TRITC-conjugated anti-rabbit, FITC-conjugated anti-mouse IgG, Percoll, DMSO, BSA, Triton X- 100, Formaldehyde, Progesterone and “AEC staining kit” were from SIGMA-Aldrich (USA).

### Sperm Preparation

Assays with human semen samples were performed in accordance with the guidelines of the Declaration of Helsinki. The semen samples were obtained from healthy donors after 3–5 days of abstinence. Only those samples exhibiting normal seminal parameters according to the WHO criteria were included in the study [58]. Spermatozoa were separated from the seminal plasma using a discontinuous (95%,47%) Percoll gradient in HAM F-10 culture medium [3]. Then, the highly motile sperm population was adjusted to 7×10^6^ cells/ml in HAM F-10 supplemented with 1% HSA (to support capacitation) or without HSA (in the case assays were performed with non-capacitated cells), and then incubated at 37°C in 5% CO_2_ on air for 4 h [2]. All the experiments were carried out with cells incubated under capacitating conditions unless otherwise specified.

### Sperm Chemotaxis and Motility Determination

The experiments were performed in a chemotaxis chamber which consists in two wells separated by a 2 mm wall, one filled with medium with or without attractants and the other with spermatozoa. The chamber was sealed with a coverslip, thus a capillary space (called bridge) was formed between both wells and over the separating wall. Across the bridge, a one dimension attractant concentration gradient was formed in the direction of the well containing the spermatozoa, which in turn, swam up over the bridge. Fifteen minutes after sealing the chamber (time necessary to stabilize the sperm distribution and the attractant gradient), the sperm movement was recorded along the fields in the middle of the bridge. Then, the sperm tracks were analyzed by video-microscopy and computer image analysis to calculate chemotaxis.

For each sperm track, the distance traveled along the X axes, (representing the attractant gradient; DX) and the Y axes (representing the absence of attractant gradient; DY) were calculated. Assuming that a chemotactic spermatozoon travels a longer distance along the X axes than in the Y axes, sperm directionality was calculated by the quotient DX/|DY|. When this value was >1, the spermatozoon was considered oriented towards the attractant well. As negative control, a culture medium without attractants was loaded instead the attractant containing solution, where ∼25% of spermatozoa swimming at random are expected to be oriented towards the well without attractants. The chemotactic responding subpopulation was considered as the difference in the percentage of “oriented spermatozoa” between the attractant solution and the negative control.

Since the chemotactic response is strongly dependent on the attractant concentration, several doses of the attractant solution should be assayed. Thus, a bell-shaped curve, typical of any chemotactic cell is observed, where at low attractant concentration there is not enough receptors stimulated, but they are saturated at high attractant concentration [59]. As a consequence, in both extreme cases the chemotaxis response is abolished and the level of “oriented spermatozoa” is similar to the basal negative control (∼25%). In contrast, at optimum attractant concentration the cells are able to sense the gradient and respond with a chemotactic movement orientation, giving a level of “oriented spermatozoa” statistically higher than the basal negative control. As in mammals such a difference is ∼10%, a high number of spermatozoa per treatment must be analyzed (minimum 300 cells), in at least three experiments.

For chemotaxis assay human spermatozoa were diluted at 4×10^6^ cells/ml and then exposed to HAM F-10 culture medium (as negative control), or the attractant gradient (10 pM P). The treatments with the inhibitors were performed 1–15 minutes (depending on the inhibitor) before the chemotaxis assays. Images were recorded at 6 Hz with the VirtualDub software (ver. 1.6.16, Avery Lee; http://www.virtualdub.org/). The sperm directionality and motility were analyzed with the ImageJ software (ver. 1.38, NIH, USA) and the MtrackJ plugin (ver.1.1.0, Eric Meijering). The percentage of “oriented spermatozoa” was calculated with the SpermTrack software (ver. 4.0, UNC, Argentina). Since chemotaxis determination can be affected by a decrease in sperm motility due to a secondly effect of the inhibitor, this parameter was additionally evaluated (see Supplementary figures S1, S2, S3).

### cAMP and cGMP Measurement

Aliquots of human spermatozoa at 15×10^6^ cells/ml, were incubated for 20 min with 100 µM IBMX (phosphodiesterase inhibitor), in the absence or presence of 10 pM P. Cells were then fixed with 1% formaldehyde for 20 min and washed twice with PBS. The pellet was resuspended in lysis reagent (from cAMP or cGMP EIA Kit) and sonicated (two times, 30 sec). cAMP and cGMP concentrations were determined according to the manufacturer's recommendations. Measurements were run in triplicate and the results comprise three independent experimental trials.

### Calcium Imaging

Spermatozoa were loaded with Oregon Green BAPTA 1-AM (12 µM in Pluronic F-127) for 30 minutes at 37°C and 5% CO_2_, then transferred to an imaging chamber, in which the lower surface was a 1% Poly-D-lysine coated coverslip. Further 30 min incubation was then allowed for the cells to adhere. The imaging chamber (volume 180 µl) was connected to the perfusion apparatus (peristaltic pump and header tank; perfusion rate 0.4 ml/min) and cells were super-fused with approximately 10 ml of fresh medium to remove unattached cells and excess dye. Images were acquired with a Nikon TE200 inverted fluorescence microscope using a 40X objetive and a Rolera XR cooled CCD camera at 8 Hz. Fluorescence intensity in the head of each cell was determined in each image and was normalized to the control value determined before stimulation with P. Data acquisition and storage were controlled by a PC running ANDOR IQ (Kinetic Imaging Ltd., Nottingham, UK) as described by [60].

### Immunocytochemistry

Spermatozoa incubated under conditions that support or not capacitation, were attached to a coverslip previously treated with poly-L-lysine 0.05 mg/ml, which was inverted over a chemotaxis chamber. Then cells were exposed to HAM F-10 culture medium, or the attractant gradient [61]. After 15 min of incubation, the coverslip was removed and the cells were fixed and permeabilized with formaldehyde 1% and 0.2% Triton X-100. The endogenous-peroxidase was blocked with 3% H_2_O_2_, and then washed with PBS. The cells were incubated with 5% BSA-PBS/0.2% Triton X-100 for 30 min at room temperature. Spermatozoa were incubated with a mouse monoclonal anti-phosphotyrosine antibody (1∶50 in 1% BSA-PBS/0.2% Triton X-100) overnight at room temperature (where in the negative control a normal mouse serum was used), after this incubation, cells were washed several times with 1% BSA-PBS/0.2% Triton X-100, followed by 2 h incubation with a biotin-conjugated anti-mouse IgG (1∶100 in 1% BSA-PBS/0.2% Triton X-100). The spermatozoa were washed several times with PBS, before treating the cells with streptavidin-peroxidase for 30 min. The cells were observed under a light microscope after staining with AEC kit. In some experiments, spermatozoa were double-stained with a rabbit polyclonal anti-Rec30 and a mouse monoclonal anti-phosphotyrosine antibody (1∶50 in 1% BSA-PBS/0.2% Triton X-100), overnight at room temperature (where in the negative control a normal serum from mouse and rabbit was used). The samples were then incubated with a TRITC-conjugated anti-rabbit IgG and FITC-conjugated anti-mouse IgG antibody. The labeling was observed under UV light with Olympus microscope equipped with a 100X/oil objective and a Olympus camera.

### Statistical Analysis

All the treatments were carried out in duplicate (unless otherwise indicated) and the results comprised three to six independent experiments. Statistical differences between treatments were determined by means of one-way ANOVA and the Tukey-Kramer tests with the SigmaStat software (SPSS, Inc, USA).

## Supporting Information

Figure S1 Effect of specific inhibitors of the AC-cAMP-PKA pathway on sperm motility and acrosome reaction. Since chemotaxis determination can be affected by a decrease in sperm motility due to a secondly effect of the inhibitor, this parameter was additionally evaluated. A–C: Sperm velocity evaluated during the chemotactic assay towards 10 pM P and percentage of motile spermatozoa after incubation with ddAdo, KH7 and KT5720, respectively. D: Percentage of acrosome reacted spermatozoa induced by 10 µM A23187 calcium ionophore after treatment with KH7 (15 minutes) and evaluated by FITC-labeled Pisum sativum agglutinin. The corresponding control value (spontaneous acrosome reaction) was already substracted. Data are expressed as the mean ± SEM. a Significant differences vs. without inhibitor (p<0.05).
(4.78 MB TIF)

Figure S2 Effect of specific inhibitors of the GC-cGMP-PKG pathway on sperm motility. Since chemotaxis determination can be affected by a decrease in sperm motility due to a secondly effect of the inhibitor, this parameter was additionally evaluated. A–C: Sperm velocity evaluated during the chemotactic assay towards 10 pM P and percentage of motile spermatozoa after incubation with ODQ, LY-83.583 and KT5823, respectively. Data are expressed as the mean ± SEM.
(1.80 MB TIF)

Figure S3 Effect of specific inhibitors of calcium signaling on sperm motility. Since chemotaxis determination can be affected by a decrease in sperm motility due to a secondly effect of the inhibitor, this parameter was additionally evaluated. A–G: Sperm velocity evaluated during the chemotactic assay towards 10 pM P and percentage of motile spermatozoa after incubation with BAPTA-AM, sEBSS culture medium with and without extracellular calcium, Nifedipine, SKF96365, TMB-8, 2-APB and Tetracaine, respectivelly. Data are expressed as the mean ± SEM. a Significant differences vs. without inhibitor (p<0.05).
(4.51 MB TIF)

Figure S4 Sperm protein tyrosine phosphorylation. Spermatozoa double-stained for (A) protein Tyr-phosphorylation (green) and (B) acrosome (red) with the corresponding phase contrast image (C). The negative controls with normal serum from the respective species are shown in the insets. * A spermatozoon showing pattern “H” and an intact acrosome.
(3.98 MB TIF)

Figure S5 Chemotactic signaling activation by cyclic nucleotides. A and B: Percentage of oriented spermatozoa (OS) towards (A) db-cAMP and (B) db-cGMP gradient. HAM and 10 pM P were assayed as a negative control and positive control, respectively. Data are expressed as the mean ± SEM. a Significant differences vs. HAM (p<0.05).
(4.47 MB TIF)

Table S1 Sequence of chemotactic signaling events. Molecules participating in sperm chemotaxis upstream or downstream the cAMP or cGMP involvement.
(0.03 MB DOC)
