# Supplementary material for: Molecular Mechanism for Human Sperm Chemotaxis Mediated by Progesterone
Source: PLoS One. 2009 Dec 8;4(12):e8211. doi: 10.1371/journal.pone.0008211 (PMC2782141; doi:10.1371/journal.pone.0008211)
Supplement: Table S1 — Sequence of chemotactic signaling events. Molecules participating in sperm chemotaxis upstream or downstream the cAMP or cGMP involvement. (0.03 MB DOC) [file pone.0008211.s006.doc]

| Molecule | cAMP step | cGMP step |
| --- | --- | --- |
| tmAC | upstream | upstream |
| PKA | downstream | upstream |
| stored Ca^2+^ release | downstream | upstream |
| IP_3_R channel | downstream | upstream |
| 1º calcium influx (SOC) | downstream | upstream |
| sGC | downstream | upstream |
| PKG | downstream | downstream |
| 2º calcium influx | downstream | downstream |
